# Re-Detectable Positive RT-PCR Test Results in Recovered COVID-19 Patients: The Potential Role of ACE2

**DOI:** 10.1017/dmp.2020.276

**Published:** 2020-07-27

**Authors:** Juan Vargas-Ferrer, Víctor Zamora-Mostacero

**Affiliations:** Facultad de Medicina, Universidad Nacional de Trujillo, Perú

**Keywords:** coronavirus, COVID-19, angiotensin-converting enzyme 2, discharged patients

Reports of re-detectable positive (RP) results of severe acute respiratory syndrome coronavirus 2 (SARS-CoV-2) RNA tests in individuals who met discharge criteria have emerged as a serious global issue in public health.^1,2^ Many studies have postulated that we are facing cases of false-negative results at the time of hospital discharge; however, phenomena of recurrence and reinfection cannot be ruled out. So far, the underlying mechanism of RP results is not well established. In this context, a closer focus on angiotensin-converting enzyme-2 (ACE2) expression in different human tissues could elucidate this issue.

The test-based strategy of the Centers for Disease Control and Prevention recommends negative RT-PCR results, from 2 consecutive respiratory specimens, collected ≥ 24 hours apart, as an alternative for discharge. For this purpose, within the early stages of the disease, the high viral load in the upper respiratory tract (URT) makes this sampling site ideal for diagnosis.^3^ Nonetheless, after admission, the positivity rates from nasopharyngeal (NP) and oropharyngeal (OP) swabs are lower than those found in sputum.^4^ Moreover, after hospital discharge, sputum swabs may remain positive even 39 days after the URT samples return negative results.^1^ Thus, as the disease progresses, the lower respiratory tract (LRT) exhibits a longer viral persistence compared to the URT.

In this instance, ACE2, an essential receptor for the SARS-CoV-2 entry, might play an essential role to elucidate RP test results. First, ACE2 is predominantly found in type II pneumocytes rather than the URT, thus, a large viral replication and load are determined by the high expression of this enzyme.^3,5^ Therefore, a slow clearance is found in the LRT, which explains why sputum swabs remain positive after the URT samples come back negative.^3^ This lead us to interpret RP test results from LRT samples, in patients with negative results from URT specimens, as false-negatives cases related to the sampling site.

Second, cases in which patients tested positive from NP or OP swabs after they were discharged with 2 negative URT samples could also be justified by the slow viral clearance in the LRT. Due to a high ACE2 expression levels in the LRT, the mucus produced in this zone could show detectable viral fragments for a longer time than the URT. At this point, the transport of mucus from the LRT to the pharynx, by the mucociliary apparatus, could account for the variability of URT swab results in some recovered coronavirus disease (COVID-19) patients. However, the reason why these detectable viral fragments are not readily degraded by ribonucleases during their transportation to the pharynx is yet to be known.

Third, ACE2 may also be an important factor to justify obtaining RP tests from gastrointestinal tract specimens. ACE2 molecules are also present in enterocytes from the ileum and colon, and, indeed, these cells express higher ACE2 levels than the URT.^5^ This is consistent with some reports in which feces swabs remain positive for a longer time than NP or OP swabs, a similar situation as the LRT samples.^1^

Taking everything into account, we strongly believe that RP results are a reflection of long viral persistence determined by high ACE2 expression in the LRT and the digestive tract. However, further studies are needed to confirm this theory.
